# Could lymphatic mapping and sentinel node biopsy provide oncological providence for local resectional techniques for colon cancer? A review of the literature

**DOI:** 10.1186/1471-2482-8-17

**Published:** 2008-09-24

**Authors:** Ronan A Cahill, Joel Leroy, Jacques Marescaux

**Affiliations:** 1 Department of Surgery, IRCAD/EITS, Strasbourg, France

## Abstract

**Background:**

Endoscopic resectional techniques for colon cancer are undermined by their inability to determine lymph node status. This limits their application to only those lesions at the most minimal risk of lymphatic dissemination whereas their technical capacity could allow intraluminal or even transluminal address of larger lesions. Sentinel node biopsy may theoretically address this breach although the variability of its reported results for this disease is worrisome.

**Methods:**

Medline, EMBASE and Cochrane databases were interrogated back to 1999 to identify all publications concerning lymphatic mapping for colon cancer with reference cross-checking for completeness. All reports were examined from the perspective of in vivo technique accuracy selectively in early stage disease (i.e. lesions potentially within the technical capacity of endoscopic resection).

**Results:**

Fifty-two studies detailing the experiences of 3390 patients were identified. Considerable variation in patient characteristics as well as in surgical and histological quality assurances were however evident among the studies identified. In addition, considerable contamination of the studies by inclusion of rectal cancer without subgroup separation was frequent. Indeed such is the heterogeneity of the publications to date, formal meta-analysis to pool patient cohorts in order to definitively ascertain technique accuracy in those with T1 and/or T2 cancer is not possible. Although lymphatic mapping in early stage neoplasia alone has rarely been specifically studied, those studies that included examination of false negative rates identified high T3/4 patient proportions and larger tumor size as being important confounders. Under selected circumstances however the technique seems to perform sufficiently reliably to allow it prompt consideration of its use to tailor operative extent.

**Conclusion:**

The specific question of whether sentinel node biopsy can augment the oncological propriety for endoscopic resective techniques (including Natural Orifice Transluminal Endoscopic Surgery [NOTES]) cannot be definitively answered at present. Study heterogeneity may account for the variability evident in the results from different centers. Enhanced capacity (perhaps to the level necessary to consider selective avoidance of en bloc mesenteric resection) by its confinement to only early stage disease is plausible although not proven. Specific study of the technique in early stage tumors is clearly essential before proffering this approach.

## Background

Advances in technological capability have made feasible the local resection of small colonic tumors by intraluminal and even transluminal endoscopy [1-4]. Although primarily now proposed for supposed benign lesions, in concept, selected germinal cancers could also be resected by these means. However, the insensitivity of preoperative radiological imaging for the detection of nodal metastases (approximately 70% of tumor-containing nodes are less than 5 mm in size [5-9]) and the inability of biopsy analysis to truly reflect the metastatic potential of the primary means that localized resection of the primary for even the earliest cancers risks either the understaging of systemic disease or the rendering of the effort redundant if formal resection becomes indicated by the full pathology of the resected specimen[10]. A reliable means of definitively establishing lymph node status peroperatively, other than en bloc mesenteric resection, would greatly increase the oncological providence of these techniques and could expand their application.

Sentinel node biopsy would seem on first principles well suited to address this breach as it fulfills a similar role in tumors of the breast and skin. This technique has also been recently proposed to accompany endoscopic dissection of early gastric cancers in order to enhance functional outcome by minimizing the extent of surgical resection [11-13]. Adjoining such a 'diagnostic laparoscopy' to an endoscopic resective technique could be justified in selected patients if the outcome of the node biopsy would permit localized excision as the definitive intervention in place of radical operation. Conversely, if the node is revealed as positive for metastases, the surgeon can confidently advocate radical operation in cases when the tumor appears confined. Synchronous laparoscopy has indeed already been advocated for the endoscopic resection of certain difficult or large polyps[14]. Furthermore, it seems likely that increasing experience with transluminal peritoneal access and intervention (i.e. Natural Orifice Transluminal Endoscopic Surgery [NOTES]) could mean that selective lymph node biopsy without abdominal wall ingress will be practicable in the near future[15]. However, lymphatic mapping in intestinal cancers is still considered controversial because of reports of varying accuracy and concerns regarding reliability and reproducibility.

To date however no comprehensive study or review has been performed from the perspective of using lymphatic mapping to facilitate minimally resective techniques for early stage colon tumors. Analyses to date have instead focused primarily on the capacity of the technique to predict recurrence risk through the upstaging of conventionally node negative disease after standard operation has been performed [16-24]. The main focus has therefore been on Stage II rather than Stage I cancers with the sentinel node biopsy and analysis being performed in addition to rather than in place of formal lymphadenectomy[25]. The aim of this review is therefore to formally interrogate the evidence base in its entirety to determine whether lymphatic mapping can, on any basis, be rationally proposed to augment the oncological propriety of localized endoscopic resection specifically for the small, early stage colon cancers that lie within its scope.

Note: Rectal cancers lie outside the premise of this review as the anatomical arrangement of the mesorectum (specifically its bulk, retroperitoneal position and lack of serosal layer) precludes against intraoperative nodal biopsy for rectal cancer. Furthermore, violation of the mesorectum may also compromise any subsequent attempt at formal oncological resection (and hence negatively impact upon patient outcome) should this be indicated by the pathology of the resected specimen.

## Methods

### Search methods for identification of relevant studies

The following strategy was used to identify relevant publications regarding experience with lymphatic mapping in human patients with colon cancer (see Table 1). Firstly, PubMed software was used to search the Medline database between 1^st ^January 1999 (the year of the earliest series published on the technique in colon cancer) and the 30^th ^July 2008. The Cochrane library (including the Cochrane Central Register of Controlled Trials) and EMBASE databases were also directly searched in a similar fashion. The following expanded Medical Subject Headings were used-'sentinel node', 'lymphatic mapping', 'colon cancer', 'colon tumo(u)rs', 'colorectal cancer/tumo(u)rs', 'large intestine' and 'gastrointestinal' (although the intent of the study was confined to colon cancer the later three search terms were included for completeness as some series report mixed cohorts). The reference list of all full publications so identified along with that of consensus papers, review articles, editorials and relevant book chapters were cross-checked for additional relevant publications. Data contained in meeting abstracts were not studied as these were judged unlikely to present sufficient detail for extraction required by our study protocol. Only English language publications were included (language bias however seems unlikely since the literature search in Pubmed and Embase did not reveal any substantial non-english reports). Finally only those studies that used vivo methodology alone for both the injection and the identification of salient sentinel nodes were analyzed (ex vivo mapping can only be reliably performed if a specimen with its complete mesentery has already been resected intact while ex vivo identification can predispose to false negative glands as redistribution of the dye along the lymphatic chain can occur in the time between injection and node harvest). Intraoperative marking of the sentinel node rather than actual excisional biopsy allowed inclusion however as clearly the intent and purpose is the same.

**Table 1:** Inclusion criteria for inclusion of publication in this review.

**Inclusion criteria**
English language publication
Full publication between 1^st ^January 1999 and July 30^th ^2008.
Human patients with colon cancer
In vivo mapping and node identification

### Assessment of methodological quality of the studies identified

Each of the studies identified was analyzed for suitability for inclusion according to the criteria laid down by QUADRAS – an evidenced base tool for the assessment of the quality of diagnostic assessment studies[26,27]. By this means it was determined whether the publication was of sufficient quality to allow inclusion in this review.

### Data extraction from included studies

All data extraction was performed by two authors (RAC and JL) with cross-checking to ensure validation. When any disparity or disagreements arose the investigators met with the third author JM as final adjudicator to resolve the debate. The fields for data capture were pre-specified before analysis of the studies identified. Data pertaining to patient demographics, technique methodology (including both surgeon experience as well as precise technical details) and sentinel node efficacy by binary classification (i.e. detection, accuracy, sensitivity and false negative rates as well as negative predictive values) for patients with colon cancer undergoing lymphatic mapping were prepared on Microsoft Excel datasheets. Data from papers that explicitly declared themselves further sub-analyses of a previous study were included with the prior publication. Studies from the same authors but which did not declare themselves to contain overlapping cohorts were analyzed separately although are flagged in the subsequent tables to indicate that this is possibility. When quantitative results were not presented and were not extractable only that data that was useful to this analysis was extracted (data not presented and impossible to extract is denoted in the tables by *ns *(i.e. 'not stated'). Otherwise the paper was excluded. In cases of mixed populations, where possible, only data relating to the in vivo mapping and biopsy of sentinel nodes in colon cancer were extracted. If not possible, the data was included with note made of the circumstances. Finally any further formal results analyses or additional hard data (rather than speculative commentary or theoretical opinion) from the Methods, Results or Discussions sections of the studies was also recorded to allow for subsequent analysis and consideration.

### Sentinel Node Performance Parameters

Where possible, 2 × 2 contingency tables were built comprising true positive (both sentinel and non-sentinel nodes involved), true negative (both sentinel and non-sentinel nodes clear), false negative (sentinel node clear but non-sentinel nodes involved). The term 'false positive' (and hence calculation of 'specificity') is not appropriate in studies regarding sentinel node in cancer because the presence of isolated macrometastases in the sentinel node confers node positivity on the patient. Nor, given the experimental nature of sentinel node biopsy and the biological uncertainty of the significance of micrometastases, is this term appropriate for use when micrometastases alone are present in the sentinel node. Instead the term upstaging is used to better reflect the standing of sentinel nodes that are immunohistochemically positive when other non-sentinel nodes are clear of disease.

The following definitions were therefore used to ascribe the performance rates of sentinel node biopsy

Detection rate – refers to the number of times a sentinel node was actually identifiable = (Number of successful attempts to retrieve a sentinel node/Number of attempts to retrieve a sentinel node)*100%.

Accuracy rate refers to the ability of the sentinel node to reflect the overall status of the lymph basin (whether positive or negative) = [(Number of correct predictions of the nodal status by sentinel node biopsy/Number of patients undergoing sentinel node biopsy)*100%].

Sensitivity refers to the number of times the sentinel node reflects the fact that disease is present in the non-sentinel nodes = (Number of patients with tumor-involved sentinel nodes/Number of patients with any lymph node containing tumor)*100%.

The false negative rate reflects the proportion of patients in whom no cancer was identified in the sentinel node but who had nodal deposits found in their non-sentinel nodes compared to the total number of those who had tumour containing metastases in non-sentinel nodes = (Number of false negative patients/Number of true positive cases + number of false-negative cases) *100%.

Upstaging rate refers to the number of cases in which sophisticated analysis of the sentinel node reveals tumor deposits that otherwise would not have been detected = (Number of patients revealing micrometastases or isolated tumor cells in the sentinel node/Number of patients classified as N0 after routine histopathological examination)*100%.

## Results

### Results of literature search

**Figure 1:**
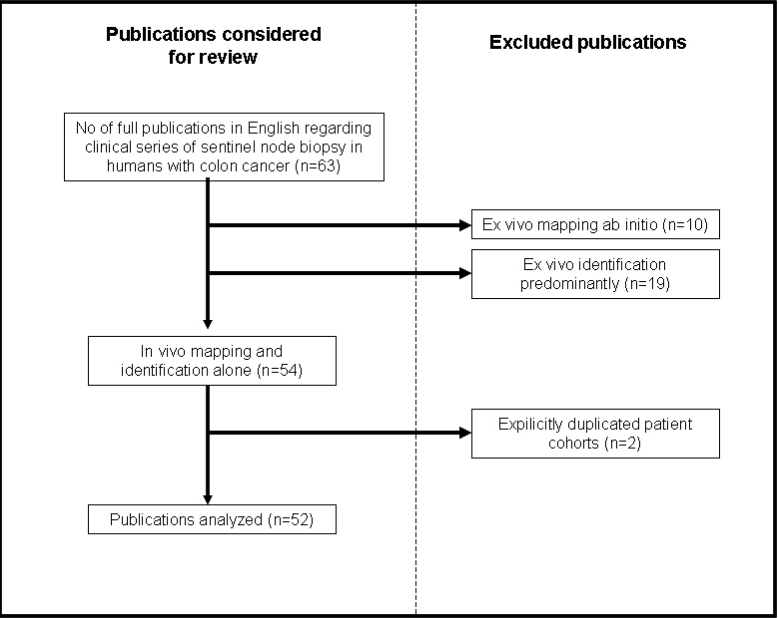
Flow chart showing the selection and exclusion of publications for this review.

There were no randomized controlled trails identified by our search methodology. Sixty-three clinical studies regarding lymphatic mapping and sentinel node biopsy for colon cancer in human patients were published in the English language during our review period of interest. Nine of these studies however actually utilized a primarily ex vivo lymph node identification technique (only the dye injection was performed in vivo and the surgeon made no attempt intraoperatively to identify any mapped nodes). These studies [28-37](along with ten additional studies that utilized a primary ex vivo technique ab initio [38-46]) were therefore excluded from further analysis (see Figure 1 and Table 2). A further two studies were explicitly declared updates or further analyses of already reported patient cohorts.

**Table 2:** Studies excluded from analysis as primarily reports of ex vivo biopsy techniques.

**Entirely ex vivo technique**			**In vivo injection but ex vivo identification of mapped nodes**		
*Lead Author*	*Year*	*Journal*	*Lead Author*	*Year*	*Journal*

Wong [28]	2001	Ann Surg	Cserni [38]	1999	Path Oncol Res
Fitzgerald [29]	2002	J Surg Oncol	Joosten [39]	1999	Br J Surg
Broderick-Villa [30]	2004	Am Surg	Merrie [40]	2001	Dis Colon Rectum
Wong [31]	2004	Ann Surg Oncol	Gandy [41]	2002	Colorectal Dis
Smith F [32]	2005	Ann Surg Oncol	Evangelista [42]	2002	Tumori
Bell [33]	2005	Dis Col Rectum	Demirbas [43]	2004	Turk J Gastroenterol
Smith J [34]	2006	Am J Surg	Krishnan [44]	2005	Indian J Gastroenterol
Yagik [35]	2007	Int J Col Dis	Van Scheltinga [45]	2006	Scand J Gastroenterol
Van Schaik [36]	2007	Eur J Surg Oncol	Faerden AE [46]	2008	Dis Colon Rectum
Stojadinovic [37]	2007	Ann Surg			

This left fifty-two studies warranting consideration for inclusion in this study [21,47-97](see Table 3). Although ten of these studies supplemented their in vivo work with additional ex vivo examination on a occasional basis (particularly in cases of failed mappings or rectal cancers), these were included in the review as their majority of cases underwent their mapping and biopsy entirely in vivo prior to specimen resection. In addition, aside from any contribution to multicenter trials, seven centers provided 27 studies to the literature base. Eight publications have come from the John Wayne Cancer Institute alone and seven have come from the McLaren Regional Center, Michigan State University alone (with a further four publications emerging from these two centers jointly). Two centers (Wake Forest, North Carolina and MD Anderson, Texas) have published two studies each while three centers (Mount Sinai, Miami Beach; Charité-University Medicine Berlin, Germany and the University Medical Center Groningen, Netherlands) have published two studies each. However, although some degree of overlap between patient cohorts is likely in successive publications that exact proportion of patients presented in duplicate was not explicitly stated and so all studies were included albeit with flagging.

**Table 3:** Publications included for this analysis along with their concluding results regarding the rates of detection, accuracy, sensitivity, false negative and upstaging as well as the false negative rates associated with lymphatic mapping and sentinel node biopsy for colon cancer.

**Lead Author**	**Year**	**Journal**	**Detection ** **Rate**	**Accuracy ** **Rate**	**Sensitivity ** **Rate**	**False Negative ** **Rate**	**Negative Predictive ** **Value**	**Upstaging ** **Rate**
Saha (a)[47]	2000	Ann Surg Oncol	99	96	91	9	Ns	18
Wiese (a)[48]	2000	Arch Pathol Lab Med	99	96	91	9	ns	ns
Waters (b)[49]	2000	Am Surg	91	100	100	0	100	6
Bilchik (c)[50]	2001	J Clin Oncol	100	100	100	0	100	50
Paramo (d)[51]	2001	Am J Surg	71	100	100	0	100	11
Wood (a)+(c)[52]	2001	Ann Surg Oncol	96	95	88	12	ns	25
Wood (c)[53]	2001	Surg Endosc	100	100	100	0	100	9
Saha (a)+(c)[54]	2001	Ann Surg Oncol	98	96	90	10	95	14
Esser [55]	2001	Dis Colon Rectum	58	94	67	33	94	7
Bendavid [56]	2002	J Surg Oncol	90	94	95	5	91	42
Paramo (d)[57]	2002	Ann Surg Oncol	82	98	93	7	97	11
Wood (c)[58]	2002	J GastroInt Surg	97	95	92	8	ns	24
Bilchik (c)[59]	2002	Eur J Cancer	97	95	91	5	ns	24
Kitagawa [60]	2002	Dis Colon Rectum	91	92	82	18	88	ns
Feig (e)[61]	2002	Am J Surg	98	79	38	62	76	8
Broderick-Villa [62]	2002	Cancer J	92	79	50	50	73	0
Tsioulias (c)[63]	2002	Am Surg	100	93	67	33	ns	15
Nastro [64]	2002	Tumori	75	100	100	0	100	ns
Bilchik (c)[65]	2003	Cancer Control	100	93	91	9	ns	14
Cox [66]	2003	Curr Surg	100	100	100	0	100	29
Bilchik (c)[67]	2003	J Clin Oncol	96	96	92	8	ns	29
Turner (c)[68]	2003	Archives Path	82	92	87	13	ns	29
Trocha (a)+(c)[69]	2003	J GastroInt Surg	98	95	84	16	93	21
Veihl [70]	2003	World J Surg	87	78	50	22	71	11
Levine (b)[71]	2003	J GastroInt Surg	92	ns	50	ns	86	ns
Saha (a)[72]	2004	Dis Colon Rectum	99	ns	88	12	ns	13
Dan (a)[73]	2004	Arch Surg	99	96	86	16	ns	5
Braat [21]	2004	Eur J Surg Oncol	94	97	80	20	92	3
Bertoglio [74]	2004	J Surg Oncol	95	92	78	22	88	ns
Read [75]	2004	Dis Colon Rectum	79	97	25	75	ns	3
Patten (e)[76]	2004	Cancer	98	89	83	17	76	20
Bertagnolli [77]	2004	Ann Surg	92	80	46	54	75	0
Saha (a)[78]	2004	Ann Surg Oncol	100	95	84	16	93	5
Saha (a)[79]	2004	Semin Oncol	100	96	92	8	ns	13
Bembenek (f)[80]	2005	World J Surg	85	ns	92	8	95	26
Codnignola [81]	2005	JJ Clin Oncol	100	ns	72	28	70	37
Dahl [82]	2005	Eur J Surg Oncol	100	92	83	17	91	ns
Bilchik (a)+(c)[83]	2006	Arch Surg	100	95	88	12	ns	23
Tuech [84]	2006	Eur J Surg Oncol	97	94	91	9	ns	ns
Saha (a)[85]	2006	Am J Surg	98	96	90	10	93	ns
Kelder (g)[86]	2006	Scand J Gastroenterol	97	93	86	14	89	33
Thomas (b)[87]	2006	Am Surg	93	20	46	54	73	5
Covarelli [88]	2006	Am Surg	95	95	86	14	ns	8
Kelder (g)[89]	2007	Int J Col Dis	97	96	89	11	93	ns
Bianchi [90]	2007	Surg End	100	95	83	17	94	9
Murawa [91]	2007	Acta Chir Belg	93	84	83	17	ns	8
Bembenek (f)[92]	2007	Ann Surg	85	86	54	46	80	21
Sandrucci [93]	2007	J Surg Oncol	100	91	92	9	ns	11
Tiffet [94]	2007	Dis Colon Rectum	92	81	80	20	74	ns
Lim (e)[95]	2008	Ann Surg Oncol	99	83	59	41	78	ns
Kusano [96]	2008	Digestive Surgery	88.5	82.6	33	67	81	ns
Quadros [97]	2008	J Surg Oncol	91	80	67	33	67	25

The definitions for each are contained in the text. All rates are %; ns denotes data neither stated within nor readily derived from the results presented within the publication. Note: several centers have published more than one series without declaring overlap of their patient cohorts – such publications are flagged by the center of origin by the inclusion of letter subscript as follows: (a) McLaren Regional Center, Michigan, USA; (b) Wake Forest, North Carolina, USA; (c) John Wayne Cancer Institute, California, USA; (d) Mount Sinai, Miami Beach, USA; (e) MD Anderson, Texas; (f) Charité-University Medicine Berlin, Berlin, Germany; (g) University Medical Center Groningen, The Netherlands. Table 4,5 and 6 also follow a similar format.

### Included studies

In total fifty two diagnostic studies (listed in Table 3) detailing 3390 patient episodes were included in this analysis. Only two studies (comprising a total of 41 patients)[52,91] however set out specifically to evaluate lymphatic mapping selectively for early stage disease. This obviously is not a sufficient cohort to draw robust conclusions about the performance of the technique in this cohort. The clinical and methodological diversity (compounded by variations in data presentation) of the remaining forty-eight studies however was such that formal meta-analysis to allow statistical analysis supplement the power was neither possible nor prudent (QUADROS data not shown). Therefore this systematic review of the literature on this subject must take the form of a narrative synthesis of the evidence base in its entirety[98].

**Figure 2:**
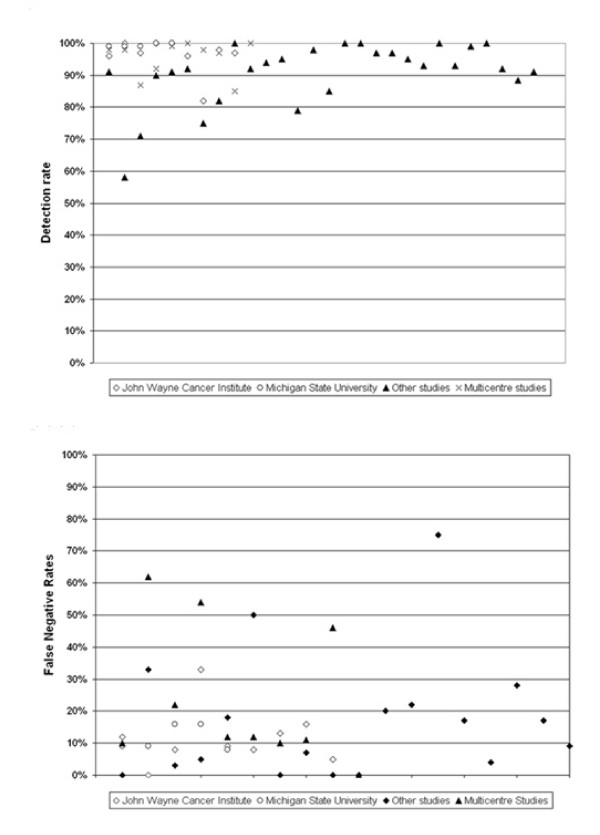
**Spread of (a) Detection rates and (b) False Negative Rates among the studies of *in vivo* sentinel node mapping in colon cancer included in this review.** In the figures, studies arising from either of the two centers with the most publications (ie The John Wayne Cancer Institute, California and the McLaren Reginal Cancer Centre, Michgan) on the topic are indicated separately to minimize any visual bias resulting from their inclusion. The remaining studies are divided into multicenter studies and all others.

Sentinel node detection rates and false negative scores are listed alongside each publication in Table 3 and displayed graphically in Figure 2. As can be seen considerable variation in both detection rates (58–100%) and, especially, in false negative rates (0–75%) is evident. To be of any clinical utility the technique would seem to require these rates to be consistently better than 90% and 10% respectively (i.e. similar to that deemed acceptable for clinical use of the technique in breast cancer). Forty-five studies present identification rates greater than this threshold (indeed 34 had rates greater than 95% while twelve identified sentinel nodes in 100% of their patients). Twenty studies reported a false negative rate of 10% or below (although eight of these studies have been published from the two centers with the most publications) and eight studies reported a false negative rate of less than 5%. While these data suggest that the technique could be apposite for the determination of operative extent, the eight and thirty studies (16% and 60% respectively of all the published experience) that fail to demonstrate such levels of efficacy raise considerable concern over its reliability and reproducibility. However the same factors that preclude meta-analysis (i.e. the considerable heterogeneity regarding variable patient cohorts and tumor characteristics as well as disparate surgeon experience and protocol differences in both the technical performance and pathological analysis) may also underlie these discordant results and will now therefore be critically analyzed in sequence. Tables 4, 5 and 6 detail the salient findings of each study by each of these criteria.

**Table 4:** Cohort characteristics with regard to baseline demographics of the patients studied.

**Lead Author**	**% Males**	**Mean or median age**	**BMI** **(kg/m^2^)**	**Non-colonic cancers included**	**% with colon cancer**	**No with colon cancer**
Saha	46	71	ns	Rectum	86	74
Wiese	44	71	ns	Rectum	70	70
Waters	ns	ns	ns	None	100	22
Bilchik	43	70	ns	Rectum	83	33
Paramo	46	72	ns	None	100	35
Wood	40	68	ns	Rectum	81	61
Wood	36	64	ns	None	100	11
Saha	ns	ns	ns	Rectum	ns	ns
Esser	55	69	ns	Rectum	84	26
Bendavid	ns	ns	ns	None	100	20
Paramo	51	70	ns	None	100	55
Wood	49	68	ns	Rectum	78	78
Bilchik	49	68	ns	Rectum	100	100
Kitagawa	71	61	ns	Rectum	21	12
Feig	73	68	ns	None	100	48
Broderick-Villa	50	63	ns	Rectum	90	40
Tsioulias	ns	ns	ns	None	100	14
Nastro	ns	ns	ns	None	100	8
Bilchik	12	64	ns	None	100	30
Cox	35	Ns	ns	None	100	17
Bilchik	48	70	ns	Rectum	85	102
Turner	53	76	ns	Rectum	86	44
Trocha	56	71	ns	Rectum	88	44
Veihl	74	75	25.3	None	100	31
Levine	55	67	ns	Stomach	74	37
Saha	48	71	ns	Rectum	83	336
Dan	49	72	ns	Rectum	88	106
Braat	ns	ns	ns	None	100	35
Bertoglio	54	ns	ns	Rectum	77	20
Read	ns	ns	ns	None	100	41
Patten	ns	64.2	ns	None	100	50
Bertagnolli	65	65	ns	None	100	72
Saha	47	71	ns	Rectum	91	52
Saha	48	71.3	ns	Rectum	80	209
Bembenek	49	45 to 83	ns	None	100	55
Codnignola	36	70.8	ns	Rectum	93	52
Dahl	57	70	ns	None	100	30
Bilchik	47	74	ns	Rectum	73	97
Tuech	ns	75.5	ns	None	100	30
Saha	48	74	ns	Rectum	82	408
Kelder	53	69	ns	None	100	30
Thomas	50	67	c. 26.3	None	100	69
Covarelli	60	70	ns	None	100	20
Kelder	ns	ns	ns	None	100	69
Bianchi	58	61	ns	None	100	22
Murawa	ns	61	ns	Rectum	48	13
Bembenek	59	67	ns	None	100	315
Sandrucci	ns	73	ns	Rectum	86	30
Tiffet	50	73	25	Rectum	75	49
Lim	48	67	ns	None	100	120
Kusano	70	70	ns	None	100	26
Quadros	36.5	56	ns	Rectum	42	22

The order and format is the same as in Table 3.

**Table 5:** Patient selection criteria along with the resulting tumor profiles in each of the studies and the mean total lymph node harvests.

**Lead Author**	**Selection criteria employed**	**Tumor Size (cm)**	**T Stage ** **(% of total)**					**T12:34 ratio**	**Conventionally Node Negative (%)**	**No of lymph nodes resected (range)**
							
			**Tis**	**T1**	**T2**	**T3**	**T4**			
Saha	None	ns	Data not given					ns	37	16 (ns)
Wiese	None	ns	0	14	22	53	11	36:63	41	16 (ns)
Waters	None	ns	Data not given					ns	27	12 (ns)
Bilchik	Early stage primary only	ns	0	26	24	50		50:50	35	15 (2–28)
Paramo	No distant metastatic disease	ns	0	12	7	81	0	7:93	29	10 (ns)
Wood	Clinically localized primary	ns	0	19	29	44	8	48:52	47	15 (2–28)
Wood	Small early stage cancers only	1.4	27	54	9	9	0	91:9	9	13 (2–20)
Saha	None	ns	Data not given					ns	40	20 (ns)
Esser	No nodal or distant metastases	ns	Data not given					ns	19.4	15 (12–16)
Bendavid	None	ns	Data not given					ns	65	ns
Paramo	No distant metastatic disease	ns	Data not given					ns	27	12 (ns)
Wood	Clinically localized	ns	Data not given					ns	26	15 (2–28)
Bilchik	Clinically localized	ns	0	25	23	46	6	48:52	43	15 (3–28)
Kitagawa	Only if curative surgery	ns	Data not given					29:71	43	24 (ns)
Feig	None	ns	7	6	23	58	6	36:64	33	13 (4–46)
Broderick-Villa	No known distant metastases	?	6	8	20	62	4	34:66	43	8 (1–17)
Tsioulias	Clinically localized only	ns	Data not given					ns	21	14 (2–21)
Nastro	None	ns	Data not given					ns	ns	ns
Bilchik	Early stage primary only	ns	20	46	14	20	0	80:20	21	14 (2–21)
Cox	None	ns	0	6	36	58	0	42:58	41	18 (4–33)
Bilchik	Early stage primary only	3.6	14	12	17	53	5	42:58	36	14 (ns)
Turner	None	ns	0	12	10	75	4	22:78	52	11 (1–42)
Trocha	No distant metastases	ns	26	12	18	42	2	56:44	38	16 (ns)
Veihl	None	4.2	0	6	9	71	12	15:83	48	21 (5–40)
Levine	No gross nodal disease	ns	Data not given					ns	ns	ns
Saha	Tumor resectable & no metastases	ns	Data not given					ns	42	ns
Dan	None	ns	17	15	13	53	3	ns	43	ns
Braat	No distant metastases, gross invasion or nodal disease	ns	0	6	20	51	23	26:74	34	9 (1–23)
Bertoglio	Stage I and II & no enlarged nodes only	ns	Data not given					ns	65	13 (6–18)
Read	Surgery with curative intent only	ns	Data not given					ns	29	14 (7–45)
Patten	No nodal or distant metastases	ns	Data not given					37:63	39	14 (ns)
Bertagnolli	Stage I, II and III only	ns	0	29	16	46	9	35:65	67	17 (ns)
Saha	None	ns	19	14	11	53	4	43:57	33	12 (ns)
Saha	None	ns	11	10	15	51	5	34:66	41	14 (ns)
Bembenek	Conventionally node negative patients only	ns	Data not given					ns	100	26 (10–59)
Codnignola	No liver metastases	ns	0	2	21	63	14	23:77	36	21 (6–47)
Dahl	No nodal or distant metastases	ns	Data not given					11:88	40	17 (4–35)
Bilchik	Potentially curable cancer with no distant metastases only	3.5 (0.2–10.5)	0	17	15	65	3	32:68	29	15 (ns)
Tuech	No nodal or distant disease	ns	0	6	9	85	0	10:90	36	20 (12–32)
Saha	None	ns	15	11	16	52	5	42:58	50	15 (ns)
Kelder	No gross nodal or distant metastases	ns	0	0	23	73	4	23:77	21	14 (ns)
Thomas	None	ns	Data not given					?	38	ns
Covarelli	None	ns	Data not given					ns	35	ns
Kelder	No gross nodal or distant metastases	ns	0	1	20	70	9	21:79	41	11 (ns)
Bianchi	No T4 or metastatic disease	ns	36	4.5	9	45	5	50:50	73	22 (8–38)
Murawa	No gross nodal or distant metastases	ns	0	15	20	63	2	37:63	41	20 (3–96)
Bembenek	None	ns	Data not given					ns	69	20 (4–79)
Sandrucci	Stage I or II only	ns	Data not given					100:0	69	9 (ns)
Tiffet	Excluded if primary unresectable.	ns	Data not given					22:78	41	18 (4–37)
Lim	No gross nodal or distant metastases	ns	0	4	26	66	4	30:70	16	13(ns)
Kusano	None	ns	0	15	73	12		88:12	77	13.5 (ns)
Quadros	Potential curable cancer, no distant metastases	8.3	0	0	14	54	31	14:86	46	19 (ns)

The order and format is the same as in Table 3.

**Table 6:** Surgeon and technical factors associated with nodal mapping, identification and pathological analysis methodology.

							**Sentinel node analysis**	
							
**Lead Author**	**Surgeon Experience sought**	**Multicentre**	**Laparoscopic resection (%)**	**Mapping agent used**	**Supplementay ex vivo identification**	**Mean/median No. of sentinel nodes (range)**	**Serial sectioning**	**Immunohist. or RT-PCR**
Saha	No	No	ns	Isosulfan blue 1%	No	1.6 (1 to 4)	Yes	No
Wiese	No	No	0	Isosulfan blue 1%	No	1.9 (1 to 4)	Yes	Yes
Waters	No	No	ns	Isosulfan blue 1%	No	ns	Yes	Yes
Bilchik	Yes	Yes	ns	Isosulfan blue 1%	No	2 (1 to 3)	Yes	Yes
Paramo	No	No	0	Isosulfan blue 1%	No	1.4 (1 to 4)	Yes	Yes
Wood	No	No	12	Isosulfan blue 1%	Yes (10%)	2 (1 to 4)	Yes	Yes
Wood	No	No	100	Isosulfan blue 1%	No	2 (1 to 3)	Yes	Yes
Saha	No	Yes	ns	Isosulfan blue 1%	No	1.7 (1 to 4)	Yes	Yes
Esser	No	No	0	Isosulfan blue 1%	No	1.7 (0 to 5)	No	No
Bendavid	No	No	ns	Isosulfan blue 1%	No	3.9 (0 to 5)	Yes	Yes
Paramo	No	No	0	Isosulfan blue 1%	No	1.6 (0 to 4)	Yes	Yes
Wood	No	No	ns	Isosulfan blue 1%	Yes (15%)	2 (1 to 4)	Yes	Yes
Bilchik	No	No	16	Isosulfan blue 1%	Yes (11%)	2 (1 to 4)	Yes	Yes
Kitagawa	No	No	0	Techneticum	No	3.5 (0 to 8)	No	No
Feig	No	Yes	0	Isosulfan blue 1%	No	2.6 (0 to 7)	No	Yes
Broderick-Villa	No	No	0	Isosulfan blue 1%	Yes (23%)	1.5 (0 to 5)	Yes	Yes
Tsioulias	No	No	100	Isosulfan blue 1%	No	1.7 (1 to 3)	Yes	Yes
Nastro	No	No	0	Technetium and blue dye	No	ns	Yes	Yes
Bilchik	Yes	No	23	Isosulfan blue 1%	No	2 (1 to 3)	Yes	Yes
Cox	No	No	0	Isosulfan blue 1%	Yes (58%)	6 (2 to 11)	Yes	Yes
Bilchik	Yes	No	ns	Isosulfan blue 1%	No	1.75 (ns)	Yes	Yes
Turner	Yes	No	ns	Isosulfan blue 1%	No	3 (ns)	Yes	Yes
Trocha	No	No	0	Technetium and blue dye	No	2.5 (ns)	Yes	Yes
Veihl	No	Yes	0	Isosulfan blue 1%	No	2 (1 to 8)	Yes	Yes
Levine	No	No	0	Isosulfan blue 1%	No	1.9 (1 to 6)	No	Yes
Saha	Yes	No	ns	Isosulfan blue 1%	No	2.1 (ns)	Yes	Yes
Dan	No	No	0	Isosulfan and fluorescein	No	2.5 (ns)	Yes	Yes
Braat	No	No	ns	Patent Blue	Yes (57%)	1.7 (1 to 4)	Yes	Yes
Bertoglio	No	No	ns	Vital Blue	No	2.9 (1 to 3)	Yes	No
Read	No	No	0	Isosulfan blue 1%	No	2 (ns)	No	No
Patten	No	No	0	Technetium and patent blue	No	3.5 (0 to 11)	Yes	Yes
Bertagnolli	No	Yes	0	Isosulfan blue 1%	No	2.1 (ns)	Yes	No
Saha	No	No	0	Technetium and Isosulfan blue	No	3 (1 to 4)	Yes	Yes
Saha	No	Yes	0	Isosulfan, Technetium and fluorescein	No	2 (1 to 4)	Yes	Yes
Bembenek	No	No	0	Patent Blue	No	2 (ns)	Yes	Yes
Codnignola	No	No	0	Patent Blue	No	2.02 (ns)	Yes	Yes
Dahl	No	No	0	Patent Blue (with radioisotope in six)	Yes (6%)	2.2 (0 to 6)	Yes	No
Bilchik	Yes	Yes	15	Isosulfan blue 1%	Yes (4%)	3 (ns)	Yes	Yes
Tuech	Yes	No	ns	Patent Blue	Yes	1.5 (ns)	Yes	Yes
Saha	Yes	Yes	ns	Isosulfan blue 1%	No	2.2 (ns)	Yes	yes
Kelder	No	No	0	Patent Blue	No	2.7 (1 to 4)	Yes	Yes
Thomas	No	No	0	Isosulfan blue 1%	No	2.1 (1 to 5)	Yes	yes
Covarelli	No	No	0	Technetium and blue dye	No	1.3 (ns)	Yes	Yes
Kelder	Yes	Yes	0	Patent Blue	No	2,3 (ns)	Yes	Yes
Bianchi	No	No	100	Patent Blue	Yes (5%)	2 (ns)	Yes	Yes
Murawa	No	No	0	Patent Blue	No	1.6 (0 to 4)	No	Yes
Bembenek	No	Yes	7	Patent Blue	No	ns	Yes	Yes
Sandrucci	No	No	ns	Patent blue and technetium	No	2.2 (ns)	Yes	No
Tiffet	No	No	0	Patent blue and technetium	No	2.6 (ns)	Yes	Yes
Lim	No	No	ns	Technetium and Isosulfan blue	Yes	4 (ns)	Yes	Yes
Kusano	No	No	62%	Indocyanine green	No	2.6 (0–5)	No	No
Quadros	No	No	0	Technetium and patent blue	Yes	3.5 (ns)	Yes	Yes

The order and format is the same as in Table 3.

### Patient Demographics (Table 4)

Ten studies gave no indication of mean (or median) patient age while eleven gave no breakdown of the patient population by gender. The age breakdown of the other studies show no especially striking data (mean age 69 years) although three studies have an unexplained clear preponderance (>70%) of males as have two of females among their cohorts. This suggests that their populations may be somewhat atypical. Only four studies present data regarding the BMIs of their patient population – a potential important discrepancy that may induce error in both detection and false negative rates as intra-abdominal obesity may obscure discolored nodes in the mesentery. Finally, only 25 (50%) studies examined colon cancer in isolation. The vast majority (26) of the other studies also included rectal cancer. These studies, despite usually declaring the proportions of each tumor studied, most often did not present result sub-analysis. While the mean number of colon cancers studied in each publication is 68, 30 studies included less than 50 of such patients while 14 comprised less than 30 patients with colon cancer.

### Tumor profiles (Table 5)

Despite the stated aim of most studies being the evaluation of the utility of lymphatic mapping for staging node negative tumors, 19 studies made no attempt to exclude patients with distant metastases or indeed evident mesenteric deposits let alone grossly involved lymph nodes. Indeed some actually specifically included such patients. Of the remainder, seven excluded only patients with distant metastases (making no further consideration of local tumour invasion or mesenteric deposits) while nine required the patient had only 'clinically localized' or 'resectable' disease to allow their inclusion. Of the twelve papers that exclude gross nodal and distant metastases, five include T4 disease while five do not profile their tumors by T-stage. Overall, at least 21 studies include T4 tumors within their cohorts. Furthermore, 25 of the studies that present such data possess high T3 and T4 to T1 and T2 ratios. Only five studies specifically consider tumor length or diameter as a factor that may affect performance parameters. Interestingly, four of these studies conclude that false negative rates are considerably more likely in patients with larger tumors while the fifth only examined this by taking 4 cm as a cut-off point for analysis. Only one study considered how tumor size may relate to the quantity of dye needed to adequately map it and found a significant positive correlation. Although the mean number of resected nodes in each study often is adequate (mean overall is in 15.3), 21 studies include patients who have had considerably less nodes than this resected in their 'definitive' operation while 25 do not state either the mean or range of the non-sentinel nodal harvest. This raises concern over the quality control mechanisms in place to ensure the standard of the oncological operation performed in these studies.

### Technical methodology (Table 6)

Only five studies specifically sought to ensure surgeon experience in the technique prior to commencing their study (although an additional nine had already demonstrated their competence in a prior publication). This is likely particularly pertinent in the ten multicentre studies, only four of whom specifically sought to ensure minimum practical experience among their participants (although a further three studies came from units that had already published experiences and so this likely evinces expertise at least among some of the contributors). Injection methodology overall was relatively similar. 45 protocols utilized an intraoperative subserosal injection of colorimetric mapping agent while three employed a submucosal injection. Twenty eight studies utilized isosulfan blue 1% in isolation while ten used Patent Blue alone and one used Vital Blue. Eight studies used a radioisotope as a mapping agent (alone in one study and in conjunction with blue dye in the others) and the majority injected this agent submucosally preoperatively by additional endoscopy. Two studies also incorporated fluorescein while one used indocyanine green. Six studies specifically included laparoscopic operations with three employing this approach exclusively. All commenting authors agreed however that the technique is easily performed regardless of operative approach and adds minimally to overall operative time[63]. The mean number of sentinel nodes found was consistently approximately two although five studies included within their range numbers in excess of double this. There was though considerable variation among how identified nodes were histologically analyzed. Four studies examined only a single section of the sentinel node while nine used neither immunohistochemistry nor RT-PCR to look for micrometastases or isolated tumor cells (one of these did however subsequently publish an additional, detailed report on their results with such techniques[99]).

Despite the fact that the variation in accuracy and sensitivity rates is frequently decried, only fifteen publications specifically included analysis of their false negative rates (see Table 7 for a tabulated summary of their conclusions). Twelve of these found that increasing tumor stage was inversely related to non-sentinel node tumors and indeed in five studies the detection rate and diagnostic accuracy was 100% among their T1 and T2 cohorts. One other study found that the presence of lymphovascular invasion was significantly associated with false negative rates but that lymph node invasion did not reach significance as a predictor (no data was shown however). The remaining study analyzing its results by tumor stage found no significance difference with either tumor stage or an arbitrarily decided lesion diameter.

**Table 7:** Tabulated summary of the specific analyses of failed or false negative analysis where such has been explicitly contained within the publication.

**Authors**	**Year**	**Comment**
Bendavid [56]	2002	The one false negative case occurred in a patient with liver metastases. Also 'evidently metastatic nodes' did not receive colourant
Paramo [57]	2002	No specific analysis presented.
Wood [58]	2002	All five false negatives occurred in T3 or T4 tumors (in one case the only positive non-sentinel node was involved by direct extension). Three occurred in 1st 30 cases
Bilchik [59]	2002	All five false negatives occurred in T3 or T4 tumors. Three occurred in the first fifty cases.
Kitagawa [60]	2002	Four false negative cases were advanced T3 and/or had massive lymph node metastases
Feig [61]	2002	Also 'several patients' (of ten) classified as false negative had 'palpable lymph nodes'
Broderick-Villa [62]	2002	Learning curve strongly associated with false negative rate (67% in first half, 32% in second half). No significant association with T-stage, LN involvement or tumor diameter > or < 4 cm
Veihl [70]	2003	Amount of dye relative to tumor size was an important predictor of identification of node. False negative more common in cases with larger nodes (4.5 cm v 3.4 cm, p = 0.09)
Bilchik [83]	2006	Of the six false negatives, four were attributable to tumor obliteration of the lymphatic channels
Saha [85]	2006	95% of patients with skip metastases were T3 or T4
Thomas [87]	2006	Two patients with liver metastases along with two others with gross mesenteric disease had false positive sentinel nodes. No relationship between BMI and disease

Kelder [89]	2006	In one of the two false negatives, the non-SLNs were involved by extra-nodal tumor invasion
Bembenek [92]	2007	Significant association with learning curve/center experience, BMI (cut-off level being 22 patients and a BMI of 25 respectively) & LVI. No significant association between detection and T stage, age, sex, vascular invasion, no of nodes, total no of nodes.
Sandrucci [93]	2007	'Skip metastases' were all correlated with 'T2 lesions with massive lymphatic involvement'
Tiffet [94]	2007	Three of 12 false negatives were in patients with direct tumor involvement of adjacent non-sentinel epicolic nodes while four were in N2 patients. False negative rate markedly lower in the subgroup with T1 and T2 tumors only. and in those with BMI < 30 kg/m^2^

### Critical analysis of studies with low performance results

The nine studies with detection rates < 90% and the thirty-two studies with false negative rates > 10% were then scrutinized from the perspectives gleaned from these analyses. Interestingly, five of the nine studies (>50%) with low detection rates also had false negative rates greater than 20% (actually 22%, 33%, 46% and 75%). Of the 43 studies with detection rates > 90%, only eleven (c. 25%) also had false negative rates greater than 20%.

Of those with detection rates < 90%, two were multicentric trials. Neither these nor six of the seven single centre studies stated they validated surgeon expertise prior to commencing patient enrollment. Furthermore each of these studies was composed of less than 60 patients. Five studies included a proportion of rectal cancers approximating 15% of the population within their study cohorts. Four studies had marked T3/4 to T1/2 preponderance (in the order of 93:3, 78:22 and 83:15 respectively). Only three studies of those with detection rates > 90% included such high proportions of locally advanced disease but one of these specifically excluded clinically apparent lymphadenopathy while the other did not contain any T4 cases. Furthermore one other study included patients with liver metastases and even obvious mesenteric deposits and direct nodal invasion by the primary while every patient in another study was conventionally node positive. Finally the patients of one report had a mean BMI above 25 kg/m^2 ^(although this was not analyzed specifically further). None of the other studies presented any data in this latter regard.

Of the sixteen studies with false negative rates above 20%, twelve presented no critical analysis of their false negative rates. Nor did any of these studies place any emphasis on surgeon experience in their stated inclusion criteria. Four studies were performed on a multicentre basis but none explicitly ensured surgeon expertise prior to commencement (in one such study the mean number of operations per surgeon was less than three) and nine studies included non-colonic tumors in between 10 and 26% of the cohort size. Furthermore one study specifically commented that tumors adherent to the retroperitoneum were included while five had T3/4 tumors accounting for approximately 80% of their patient cohorts. Three had a mean tumor size of greater than 4.2 cm with one concluding that its false negatives case were associated with significantly bigger tumor sizes (7.2 cm in the false negative cases versus 5.2 cm mean size overall). One additional study also found a strong trend in favor of an association between false negative rate and larger tumor sizes (4.5 cm v 3.5 cm, p = 0.09). Two studies had a mean number of resected lymph nodes of eight and nine respectively while at least twelve studies included some patients with less than ten nodes in their resected specimens (eight including some with five or less nodes examined). Two others presented no mean data on this subject and three presented no range. Four did not serially section the sentinel node while six did not employ immunohistochemistry or RT-PCR.

With respect to the fifteen publications with false negative rates between 10 and 20%, only three studies included specific analysis of their rates. Of the fifteen, two were multicentric trials and both sought to ensure surgical expertise and had false negative rates each of 11 and 12%. Nine studies included rectal cancers in their cohorts (in between 9% and 74% of their cohorts). Four studies had no explicit exclusion criteria while six sought only to exclude distant disease deposits. No study provided any data on patient BMI. Only one study specifically excluded T4 disease. Eight had a significant (>60%) T3/4 preponderance (being >70%). The mean number of resected lymph nodes was greater than ten in fourteen. Four studies included patients with less than this number while the one presented no data regarding the range. Two studies did not employ serial sectioning of the identified sentinel nodes and two did not utilize immunohistochemical or RT-PCR methods of examination.

## Discussion

Sufficient lymph basin resection is an oncologic sine non qua of operation with curative intent for solid organ malignancies as nodal status remains the primary portent of prognosis and adjuvant therapy prescription. For colon cancer, staging propriety by convention demands that this equate to en bloc resection of the entire mesenteric lymphatic delta. While the manner of performance of standard resectional operation for colon cancer by laparoscopy or laparotomy means that the extent of access is already determined (and so supplementing bowel resection with full mesenteric resection is readily facilitated) this is not for case for endoscopic resectional techniques. Therefore these techniques are currently limited to the address of benign lesions or neoplastic disease with minimum likelihood of lymphatic dissemination. A minimally invasive means of reliably confirming the lymph node status could greatly enhance the oncological propriety of these approaches and extend their indications towards their actual technical capacity. While it is clear that sentinel node biopsy is not indicated to minimize the extent of therapeutic lymphadenectomy in colon cancer (i.e. the resection of nodes containing tumor deposits), it could theoretically have a role in helping select those with truly early stage disease for endoscopic resection.

Furthermore, although it is often considered that adjoining mesenteric lymph node dissection to the intestinal resection impacts little on the patient undergoing conventional oncological operation for colon cancer [100-103], this assumption does not necessarily stand up to close scrutiny. The high-tie of the arterial inflow arcade necessary to adequately perform mesenteric nodal dissection however segues wider longitudinal intestinal margins than would otherwise be necessary for local clearance purposes alone. The negative impact of wide dissection and resection may include post-operative bowel dysfunction, nerve plexus praxia or adjacent organ injury [104-106]. Therefore, tacit knowledge, as well as some prior clinical investigation [107-109], would suggest that minimizing the extent of mesenteric dissection could pay dividends for the patient even if standard surgical access is employed. These considerations have however been lost amid the concerns regarding the efficacy of lymphatic mapping in this disease and the current emphasis on ensuring adequate staging by resection of considerable 'minimum' numbers of lymph nodes [110-112].

The focus of sentinel node biopsy in colon cancer has therefore been on upstaging conventionally node negative patients *after *conventional radical operation[113]. This means that false-negative sentinel nodes do not impose any negative effect on the patient but also has conferred a priori license to investigators to modify the technique and broaden the patient cohort. To propose that it could be used a means of predicting a negative basin and obviating mesenteric dissection places great emphasis on ensuring that the technique's rationale in colon cancer is biologically sound and that methodological discrepancies are eliminated. However the literature base to date contains multiple un-standardized variables and so assessment of the true utility of lymphatic mapping when applied selectively to early stage disease is considerably confounded. The high accuracy rates in some studies do at least suggest that its concept is biologically tenable (indeed of those centers that have published multiple series, four conclude strongly in favor of the reliability of the technique) and that methodological variability may underlie the discordant results (as they do for the same technique in malignancies at different sites). However, this heterogeneity also confounds any attempt at meta-analysis to selectively extract data regarding the reliability of sentinel node biopsy selectively in early stage cancer. A similar conclusion was also reached by another group performing a detailed statistical systematic review of the literature from another perspective[114]. Although their review contains significant differences in study inclusion and exclusion criteria to ours, it is interesting that they nonetheless concluded that the technique had a 9% median risk of a false negative result in comparison to an 8.4% median risk for the technique in breast cancer. However, the general tendency to pool results in the reporting of studies frustrated their attempt to relate T-stage to sensitivity.

The first evident confounder to obtaining clarity regarding the accuracy of the technique is the number of publications emanating from single centers. Although it seems likely that these seven centers (who together have contributed over half of the published evidence base) have overlapped their patient cohorts at least to some extent in successive publications, the exact proportions is rarely explicitly declared. Equally however it cannot be assumed that these studies entirely overlapped their experiences and so to ensure fairness and transparency every study meeting the inclusion exclusion is included in this study with those coming from the same center being flagged in the Tables. The next main obstacle within each publication complicating deliberation of the technique's applicability for early stage colon cancer is the marked contamination of rectal cancers throughout the evidence base. The consequences of doing so without presentation of complete subgroup analyses gives an artificial impression of reduced feasibility and accuracy rates overall because lymphatic mapping is clearly more arduous and less reliable in this site[115]. Furthermore, specific account of tumor size and mural penetration in colon cancer is too often lacking for any firm conclusions to be made regarding the efficacy of selectively mapping early stage disease. The main inclusion criterion to date is primarily the requirement for operation for symptomatic colon cancer despite the fact that the disease tends to present late (often at stage when curative surgery is not possible and adjuvant treatment is already indicated regardless of the nodal status). An elongated diameter of the cancer may also mean that different segments of the tumor map to different nodes as watershed areas are crossed. Furthermore, direct spread through the bowel wall may compress and alter the original lymphatic flow and so cause the mapping agent to deviate away from the original first order draining nodes. Extensive nodal involvement, including total destruction of the nodes by metastases, may also compromise the identification of true sentinel nodes[22,116]. Such concerns have been confirmed in the analyses presented here as well as in ex vivo experiences of mapping colon cancer and indeed in other tumors [117-119].

Surgeon expertise and experience has already been determined a major feature for lymphatic mapping in breast cancer[120] and seems likely to also be one for colonic mapping (a point made clear in one study attempting ex vivo biopsy that found many of their failed mapping were associated with intraluminal dye injection)[39]. The relationship between low detection rate and higher false negative rate is a likely related phenomenon while lack of technical proficiency may in addition compound any technical difficulties associated with adverse patient factors (such as BMI or previous laparotomy) to further undermine the performance of the technique. A specific learning curve can also certainly be expected if the surgeon looks for the nodes intraoperatively. However the lack of specific inclusion criteria for this seems to suggest that many of the studies published to date are in fact reflections of expertise acquirement rather than reports of technique reliability. The timing of the vital blue dye injection prior to resection may also be a crucial issue. A prolonged latency in identifying the node (depending on the intraoperative situation) may allow overflowing of the dye and potentially complete decoloration of the first stained node. Also the discrepancy in standardizing nodal analysis between the reports is concerning as standard assessment based on hematoxylin and eosin staining of one level of a paraffin-embedded block reportedly can miss as many as 33% of metastases[121]. While micrometastatic disease may of itself be of considerable clinical importance [115], these cells in sentinel nodes may also confer significance by their prediction of the presence of non-sentinel nodal metastases. Finally the numbers within the nodal harvest rates of many studies raises serious concerns over quality assurance utilized given that this aspect is of utmost importance when a novel technique is compared to established practice.

Certain groups have however clearly managed to overcome confounding factors of the technique and consistently obtain negative predictive values of similar quality to those that currently justify conservative resection fields in other specialties. It is perhaps no surprise that these investigators tend to fastidiously analysis their false negative results as did the pioneers of the technique in breast cancer and melanoma. On the other hand, other authorities have not hesitated to declare the technique in colon cancer either invalid or of dubious clinical value[40,122] on the basis of their own experience and perception of the evidence base. Most of the latter group did not however present data detailing their efforts to examine the potential biological reasons behind their results. As much therefore as it is clear that certain proponents in both single centre and multicentre trials can perform sentinel node biopsy with high accuracy for colonic malignancy, it is equally clear that others cannot. The fact that advanced endoscopic resection is at present only wrought in supraspecialized centers may mean however that only such selected, expert departments need attempt to investigate, validate and standardize the performance of sentinel node biopsy to accompany it. Also, the need for high detection and accuracy rates implicit if the results were going to impinge on patient care could drive efforts to develop methods to improve the yield of lymphatic mapping by use of additional or alternative dyes (perhaps fluorescent markers[73,123] or radioisotopes[69,78]) as have been used for other cancers [124-126].

The general tendency of a relationship between advancing stage an increased likelihood of metastases being present in non-sentinel nodes also supports the basic contention that lymphatic mapping in colon cancer may be particularly efficacious in germinal cancers – a perspective made particularly compelling by the inherent suitability of early stage disease for truly minimally invasive resective techniques. The lesions that could be resected by endoscopic means are by definition smaller and therefore likely confined to a single lymphatic delta. Furthermore T3 or T4 disease identifiable by staging is not feasibly resected endoscopically and so the tumor stages with the highest frequency of being node-positive are excluded. The fact that 20–40% of patients with T3/4 lesions but without demonstrable lymph node metastases actually die of their disease also supports exclusion of these patients from non-radical operation. De facto confinement of the patient cohort to T1 and T2 stage disease (perhaps by including adjunctive staging measures such as endoscopic ultrasound[127]) may therefore obviate some of the main confounders of lymphatic mapping and potentially allow proffering of localized tumor resection as definitive surgery. However the lack of specific study or discriminating subgroup analysis by T-stage means that this aspect of lymphatic mapping in colon cancer has yet to be definitively explored and so this remains speculative.

If sentinel node biopsy is ever to be used as means to alleviate mesenteric resection in cases that are truly node negative, consideration must be given to the cases where the sentinel node is positive or indeed falsely negative. In the former case, subsequent radical lymphadenectomy should still be performed. Ideally therefore the sentinel node analysis should be performed intraoperatively to allow direct progress to the definitive excisional surgery (whether of the primary alone or of the traditional extended operation). Considerable precedent exists for such analysis in breast cancer [128-131] and it is even possible that additional innovative technology may allow for in situ virtual biopsy of the salient nodes[132,133]. In the situation that the sentinel node is falsely negative, suspicious features present in the resected specimen may encourage revisional surgery in some cases. Although re-intervention after a previous laparotomy may be difficult, the risk of added morbidity because of a prior minimally invasive 'cherry-picking' of sentinel nodes is likely to be less. However any true consideration of the risk involved needs balancing with an accurate measure of the potential benefit of avoiding mesenteric resection. The consequence of missed positive lymph nodes is mainly one of potential understaging and hence depriving the patient of systemic chemotherapy. However it should also be realized the added benefit of chemotherapy over standard surgery for T1 and T2 lesions is low and perhaps that the clinical significance of minimal nodal disease in these patients is lower than that of more advanced T-stage (as is the case for early gastric cancer[134])[135,136]. The actual clinical risk of detriment due to mesenteric lymphadenopathy per se is also low[85]. It is worth however here emphasizing again that these deliberations only apply to colon cancer and not its counterpart in the rectum. The anatomic characteristics of the mesorectum (namely its bulk, extraperitoneal situation and lack of a serosal layer) mitigate against selective sentinel node biopsy in isolation for rectal cancer however as does the fact that transgression of its planes may complicate any subsequent total mesorectal resection in cases of nodal positivity and so impact upon the potential for curative resection. Finally the consequences of missed nodal disease in the pelvis and, therefore the occurrence of pelvic recurrence, may be catastrophic for the patient.

## Conclusion

Sentinel node mapping could never substitute for a properly performed oncologic colorectal resection when this is indicated. The concept however that lymphatic mapping may have sufficient capability to provide the oncological proprietary for the curative surgery for early stage cancers without en bloc mesenteric resection seems biologically plausible but cannot yet be definitively judged given the lack of clarity and consistency in the literature to date. Specific study of the technique in those early stage tumors likely to be selected for endoscopic resection is clearly therefore essential before this approach can be considered in clinical practice.

## Competing interests

The authors declare that they have no competing interests.

## Authors' contributions

RAC conceived of the study, participated in the study design and performance (performed the initial literature review and analysis) and composed the manuscript. JL contributed to the study design and performance and guided the manuscript composition. JM conceived of the study and participated in its design and coordination. All authors read and approved the final manuscript.

## Pre-publication history

The pre-publication history for this paper can be accessed here:


